# Mapping the Evidence for Opioid-Mediated Changes in Malignancy and Chemotherapeutic Efficacy: Protocol for a Scoping Review

**DOI:** 10.2196/38167

**Published:** 2023-05-22

**Authors:** Jonathan E Constance, Mary M McFarland, Tallie Casucci, Michael W Deininger, Elena Y Enioutina, Kathleen Job, Richard S Lemons, Carol S Lim, Robert M Ward, Venkata Yellepeddi, Kevin M Watt

**Affiliations:** 1 Division of Clinical Pharmacology Department of Pediatrics University of Utah Salt Lake City, UT United States; 2 Spencer S. Eccles Health Science Library University of Utah Salt Lake City, UT United States; 3 J Willard Marriott Library University of Utah Salt Lake City, UT United States; 4 Versiti Blood Research Institute Milwaukee, WI United States; 5 Division of Hematology Department of Medicine Medical College of Wisconsin Milwaukee, WI United States; 6 Division of Hematology and Oncology Department of Pediatrics University of Utah Salt Lake City, UT United States; 7 Department of Molecular Pharmaceutics College of Pharmacy University of Utah Salt Lake City, UT United States

**Keywords:** opioid, opioid receptor, drug, cocaine, crack, prescription opioid, opium, war on drug, cancer, chemotherapy, drug-drug interaction, malignancy, treatment, oncology, tumor, survival, antineoplastic, cancer cell, scoping, chemotherapeutic, librarian, library science, antineoplast, cancer cell survival, cancer cell growth, addict, addiction

## Abstract

**Background:**

Numerous reports contend opioids can augment or inhibit malignancy. At present, there is no consensus on the risk or benefit posed by opioids on malignancy or chemotherapeutic activity. Distinguishing the consequences of opioid use from pain and its management is challenging. Additionally, opioid concentration data is often lacking in clinical studies. A scoping review approach inclusive of preclinical and clinical data will improve our understanding of the risk-benefit relationship concerning commonly prescribed opioids and cancer and cancer treatment.

**Objective:**

The aim of the study is to map diverse studies spanning from preclinical to clinical regarding opioids with malignancy and its treatment.

**Methods:**

This scoping review will use the Arksey six stages framework to (1) identify the research question; (2) identify relevant studies; (3) select studies meeting criteria; (4) extract and chart data; (5) collate, summarize, and report results; and (6) conduct expert consultation. An initial pilot study was undertaken to (1) parameterize the extent and scale of existing data for an evidence review, (2) identify key factors to be extracted in systematic charting efforts, and (3) assess opioid concentration as a variable for its relevance to the central hypothesis. Six databases will be searched with no filters: MEDLINE, Embase, CINAHL Complete, Cochrane Library, Biological Sciences Collection, and International Pharmaceutical Abstracts. Trial registries will include ClinicalTrials.gov, Cochrane CENTRAL, International Standard Randomised Controlled Trial Number Registry, European Union Clinical Trials Register, and World Health Organization International Clinical Trials Registry. Eligibility criteria will include preclinical and clinical study data on opioids effects on tumor growth or survival, or alteration on the antineoplastic activity of chemotherapeutics. We will chart data on (1) opioid concentration from human subjects with cancer, yielding a “physiologic range” to better interpret available preclinical data; (2) patterns of opioid exposure with disease and treatment-related patient outcomes; and (3) the influence of opioids on cancer cell survival, as well as opioid-related changes to cancer cell susceptibility for chemotherapeutics.

**Results:**

This scoping review will present results in narrative forms as well as with the use of tables and diagrams. Initiated in February 2021 at the University of Utah, this protocol is anticipated to generate a scoping review by August 2023. The results of the scoping review will be disseminated through scientific conference proceedings and presentations, stakeholder meetings, and by publication in a peer-reviewed journal.

**Conclusions:**

The findings of this scoping review will provide a comprehensive description of the consequences of prescription opioids on malignancy and its treatment. By incorporating preclinical and clinical data, this scoping review will invite novel comparisons across study types that could inform new basic, translational, and clinical studies regarding risks and benefits of opioid use among patients with cancer.

**International Registered Report Identifier (IRRID):**

PRR1-10.2196/38167

## Introduction

This project stems from a multidisciplinary team approach to better understand the consequences of drug interactions known to occur commonly in the management and care of patients with cancer. In contrast with typical scoping review methodology comprised exclusively of clinical data, this study incorporates a more comprehensive mapping of the literature, inclusive of preclinical data. In many clinical studies, it is difficult or impossible to disambiguate patient outcomes related to opioid use from the consequence of pain or pain management. This is because opioid use is often the measure by which pain is assessed. Therefore, our approach will enable a new perspective on an old and controversial problem.

Opioid analgesics are among the most frequently administered drugs for patients with cancer, and their use often coincides with active chemotherapeutic regimens [1]. The intended target for opioid analgesics is the µ-opioid receptor (µOR) expressed within the central nervous system [2]. However, the µOR is also present in diverse cancer types [3]. In cancer cells, µOR activity can stimulate intracellular signal cascades that regulate processes of cell survival and death and can coincide with the same signal cascades triggered by cytotoxic chemotherapy [3-12]. Some clinical studies for specific cancer types have identified the µOR as a negative prognostic factor and associated with chemotherapy resistance [3,13-23].

Paradoxically, opioids have demonstrated potential to both augment and inhibit cancer cell growth and survival as well as chemotherapeutic activity. For some types of cancers, opioid use is linked to metastasis, proproliferative effects, and decreased patient survival, while in other cancer types, opioids are associated with improved cancer cell killing and improved patient outcomes [16-19,21-47]. At present, there is no consensus on the risk or benefit posed by opioids in the context of malignancy and its treatment.

While multiple clinical studies have been conducted to evaluate the relationship between malignancy and its treatment with the µOR and analgesic opioid use, interpreting the risks or benefits of opioid use on tumor growth remains challenging and often confusing. Public response to some literature reports has led to surges in demand for opioids as potential antineoplastics. In contrast, other published data have led to concern that opioids will be withheld in the presence of suffering for fear of promoting malignancy [11,48,49]. Therefore, an improved understanding of the effects routinely prescribed opioids can have on any neoplasm, cancerous lesion, or malignancy and its treatment is an urgent public health need. A scoping review approach can help meet this need by organizing the existing clinical and preclinical evidence in a manner suitable to better distinguish clinically meaningful patterns and identify key knowledge gaps.

By examining both clinical and preclinical evidence together, 3 major challenges found in interpreting the clinical literature can begin to be addressed. First, clinical studies typically have a small n and focus on a specific cancer subtype, and among these, they differ in experimental design, intervention (eg, type, timing, and route of opioid administration), and patient population. Second, clinical opioid concentration data are lacking. Opioid concentration data are critical to establish the consequences of opioid use in the context of malignancy. As opioids are characterized by high inter- and intraindividual pharmacokinetic (PK) variability, the common practice of using opioid dose as a surrogate is a highly flawed means of estimating tumor tissue concentrations [50,51]. Finally, clinical studies seeking to assess the risks and benefits of opioids related to disease progression are frequently confounded by the role of pain and pain management on patient outcomes [41,52]. Preclinical studies have the potential to fill gaps in knowledge regarding opioid effects for certain cancer models at known concentrations and are not influenced by pain.

It is to be expected that the relationship between heterogeneous forms of cancer, µOR expression, and the clinical use of various analgesic opioids (routes of administration, formulations, and patterns of use) will be complex. Nonetheless, a scoping study approach can map evidence from both clinical and preclinical studies that will augment one another in the detection of meaningful associations between analgesic opioid exposure and the potential risks or benefits to patients with cancer.

Our objective is to map preclinical and clinical study data on therapeutically relevant concentrations of opioids effects on tumor growth or survival, or alteration on the antineoplastic activity of chemotherapeutics. We will map and evaluate published evidence regarding (1) opioid concentration data from human subjects with cancer, yielding a “physiologic range” to better interpret available preclinical data, (2) patterns of opioid exposure and disease and treatment-related patient outcomes, and (3) the influence of opioids and µOR activity on cancer cell survival and growth, as well as changes in susceptibility to clinically used chemotherapeutics. Apparent contradictions and clinically relevant knowledge gaps concerning the risks or benefits of analgesic opioid use in the context of malignancy can be better understood and clarified by using a systematic approach to catalog and examine existing literature from a pharmacologic perspective.

A search for existing evidence reviews or protocols on topic has been conducted (TC) in PROSPERO and MEDLINE (Ovid) on February 6, 2021. None were identified by the team as being on topic.

Prior to developing the scoping methodology protocol, a pilot study was undertaken to evaluate existing clinical and preclinical data concerning the effects of analgesic opioids on malignancy and its treatment (JEC). The central research question proposed for this study has been sought before and remains the source of much controversy. However, as the pilot study demonstrated, harnessing both clinical and preclinical information can bring into focus central concepts, such as the use of physiologic versus supraphysiologic opioid concentrations and biologic context. These concepts are often absent in the broader discussion of opioids and malignancy yet essential to solving some of the seeming contradictions. As this prestudy data, using an inclusive approach, revealed novel and informative patterns, we anticipate, a full scoping review will provide a new framework to view existing evidence and therefore, its interpretation.

The pilot study used MEDLINE (Ovid) for years 1946-2021, titles and abstracts were screened for inclusion (full publication). References of included studies were additionally assessed for relevancy and potential inclusion. Study data were collected and managed using Excel (Microsoft Corp) and GraphPad Prism (version 9; GraphPad Software, Inc). Data elements extracted were manuscript information (journal, title, authors, and year), study type (clinical [human subjects], including ex vivo), preclinical (in vivo [animal model] or in vitro), malignancy (type, subtype, model, or cell line), ligand (µOR agonist [ie, most analgesic opioids], antagonist, and partial agonist) and concentration information (eg, IC_50_), any coinciding antineoplastic treatment, and predominant effect reported (ie, promotion or inhibition of tumor growth or chemotherapeutic activity). For ex vivo or in vitro data, “clinical” concentrations of µOR agonists were based on human subject studies reporting maximal peak concentrations experienced by adult patients with cancer receiving opioids as part of the standard of care: morphine (≤350 nM), fentanyl (≤10.8 nM), methadone (≤3.2 µM), and oxycodone (≤320 nM), and antagonists: nalmefene, methylnaltrexone, naltrexone, and naloxone (≤1 µM).

**Figure 1:**
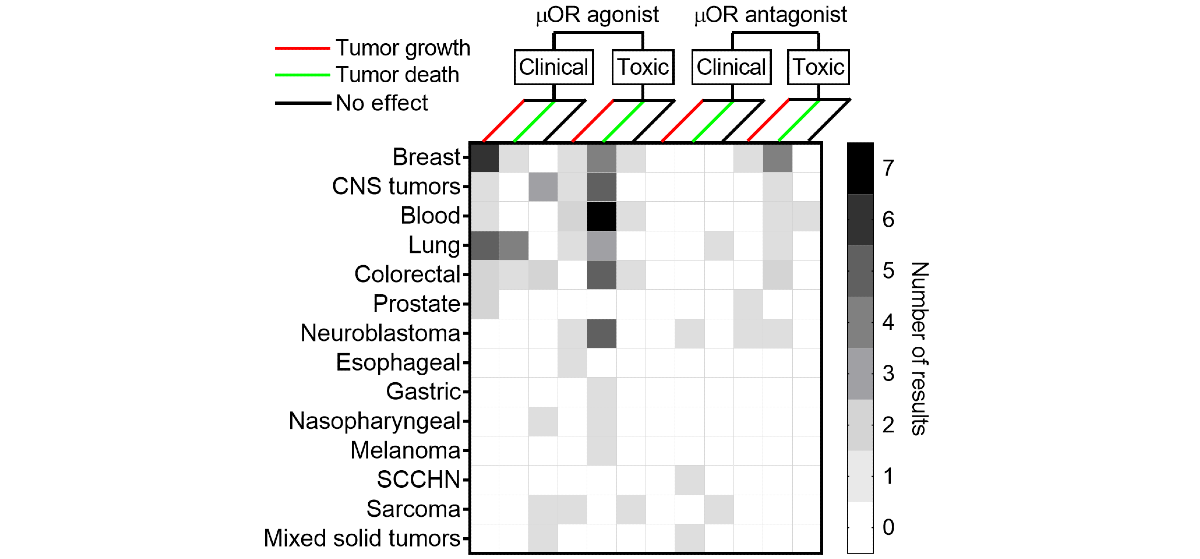
Summary data from in vitro, in vivo, and clinical studies of opioids at “clinical” or “toxic” concentrations grouped by cancer type. µOR: µ-opioid receptor; CNS: central nervous system; SCCHN: squamous cell carcinoma of the head and neck.

Data were extracted from 94 studies found to meet the pilot study criteria. From each study, findings were categorized as “tumor growth,” “tumor death,” or “no effect.” A given study could report multiple findings, for instance, morphine (µOR agonist) could be found to stimulate cancer cell proliferation in a breast cancer cell line, while naloxone (a µOR antagonist) may be found to induce cancer cell apoptosis. Aggregated findings of opioid-associated effects on cancers are presented in Figure 1 [7-10,12,17-23,30,31,33-40,42,46,52-121].

The following conclusions have been drawn:

- The magnitude of the literature makes a scoping approach feasible, with fewer than 100 total clinical and preclinical studies found to meet the pilot study criteria for inclusion.
- Clinical studies evaluating opioid effects on malignancy are typically retrospective, and opioid concentration data among patients with cancer are scant.
- Preclinical studies often use opioids at concentrations exceeding those considered safe for humans. Additionally, preclinical studies which use opioid concentrations reflecting physiologically relevant concentration ranges for humans, on the basis of comparison with existing clinical concentration data, can offer important dose-response information but pose challenges for clinical interpretation.
- Important patterns emerged, including confirming the hypothesis that for cancer tissue, opioid concentration is a critical dimension to understand the effect. Clinically relevant µOR agonist concentrations were often associated with cancer growth, while “toxic” or supraphysiologic concentrations were associated more often with cancer cell killing. Concentration is a critical, but often overlooked, dimension of opioid influence on malignancy and emphasizes the need for PK data in patients with cancer.
- The expression profiles of the µOR are poorly understood for many cancer types.

## Methods

### Study Design

We will conduct our scoping review with guidance from the latest version of the *JBI Manual for Evidence Synthesis* [122]. Using the framework as outlined by Arksey [123] and expanded by Peters [124], we will conduct our scoping review with Arksey’s [123] six stages: (1) identifying the research question; (2) identifying relevant studies; (3) study selection; (4) charting the data; (5) collating, summarizing, and reporting the results; and (6) expert consultation involving oncologists, pain management specialists, and basic and translational scientists with expertise in pharmacology and cancer biology. Consultations will be held to inform the process of project conception, protocol development, study design and conduct, and finally, the interpretation of data, including discovery and description of key knowledge gaps [123,124]. For transparency and reproducibility, we will adhere to the Preferred Reporting Items for Systematic Reviews and Meta-Analyses extension for Scoping Reviews (PRISMA-ScR) reporting guidelines in reporting our results [125].

We will use Covidence (Veritas Health Innovation), a web-based systematic reviewing platform, to screen and select studies. Citation management and duplicate detection and removal will be accomplished with EndNote (Clarivate Analytics). Research Electronic Data Capture (REDCap) tools hosted at the University of Utah will be used for data capture and charting [126].

### Literature Searching

#### Overview

A librarian (TC) will develop and translate search strategies for the web-based databases using a combination of keywords and controlled subject headings unique to each database. Peer review of the strategies will be conducted by an information specialist (MMM) using the Peer Review of Electronic Search Strategies guidelines [127]. Preliminary searches by 2 reviewers (JEC and VY) resulted in pilot data of 94 studies, which will be used by the librarian to harvest search terms.

#### Electronic Sources

We will search the following databases: MEDLINE (Ovid) 1946-2022, Embase 1974-2022, CINAHL Complete (Ebscohost) 1937-2022, Cochrane Library 1898-2022 including CENTRAL 1898-2022, Biological Sciences Collection (ProQuest) 1946-2022, and International Pharmaceutical Abstracts (Ovid) 1970-2022.

#### Other Sources

Trial registries will include ClinicalTrials.gov, Cochrane CENTRAL Register of Controlled Trials (Wiley), International Standard Randomised Controlled Trial Number registry, European Union Clinical Trials Register, and World Health Organization International Clinical Trials Registry.

No gray literature will be designated for searching. For studies meeting inclusion criteria, references will also be evaluated for relevancy and potential inclusion.

### Study Selection (Eligibility Criteria)

#### Inclusion Criteria

##### Participants

Patients in clinical studies diagnosed with any type of malignancy will be included.

##### Concept

We will assess the use of therapeutic opioids or assessment, direct or indirect, of opioid receptors or any other proposed mechanism for opioid action.

##### Context

For preclinical studies, any investigation involving any cancer cell lines, xenografts, ex vivo specimens, or relevant in vitro models of cancer related to clinically used opioids, opioid receptors, opioids purported to act upon, bind to, or influence cell processes via known or unknown targets will be included.

For clinical studies, patients diagnosed with, or survivors of, any type of cancer may or may not have received opioids at any time. Studies including patients with cancer but without opioid exposure will be included, if the study concerns biomarkers, outcomes, or other parameters related to opioid receptor or opioid influence on a malignancy or its treatment.

English-language studies will be preferred. We will chart non–English-language studies meeting or appearing to meet inclusion criteria.

#### Exclusion Criteria

Conference abstracts, studies exclusive to the topic of opioid addiction and abuse and not involving a cancer diagnosis or containing opioid concentration data, and those exclusive to the topic of pain and pain management and do not involve a cancer diagnosis or contain opioid concentration data will be excluded.

### Team

Reviewers (JEC, VY, and KJ) will screen, independent of each other, the titles and abstracts of search results. Reviewers will then review the full text, also independent of each other’s vote. Consensus will be reached through discussion among the reviewers. Any ties will be decided by a fourth independent reviewer (KMW, CSL, EYE, or RSL).

### Quality Assessment

In compliance with scoping review methodology, no quality assessment of included studies will be performed as our goal is to rapidly map the literature.

### Data Extraction: Charting the Data

Study data will be collected and managed using REDCap tools hosted at the University of Utah [126]. REDCap is a secure, web-based application designed to support data capture for research studies, providing (1) an intuitive interface for validated data entry, (2) audit trails for tracking data manipulation and export procedures, (3) automated export procedures for seamless data downloads to common statistical packages, and (4) procedures for importing data from external sources [126].

**Figure 2:**
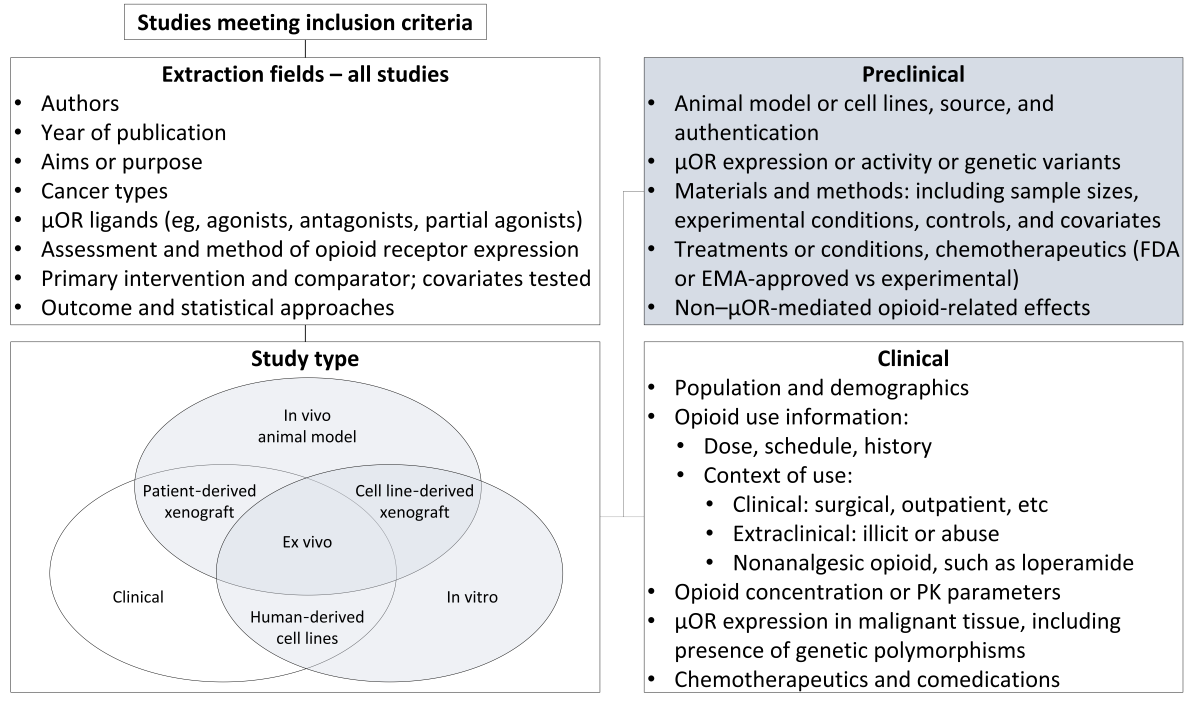
Data extraction and charting. Studies concerning the influence of clinically used opioids on malignancy and its treatment do not align with a strictly “clinical” versus “basic” science dichotomy. Capturing data elements from across the spectrum of study types will provide a more holistic view to benefit the goals of this scoping review and distinguishes it from other reviews. µOR: µ-opioid receptor; EMA: European Medicines Agency; FDA: U.S. Food and Drug Administration; PK: pharmacokinetic.

Extraction fields were developed based on the initial prescoping review pilot study to adequately address the primary research question (Figure 2). This includes extraction and charting of general manuscript information as well as adopting strategies to facilitate disaggregation of studies dependent upon data type (eg, clinical vs preclinical) for analysis.

### Analysis of Evidence

#### Overview

A descriptive analysis will be used across all studies (eg, retrospective, clinical trial, and in vitro) and be inclusive of reported outcomes. For each cancer subtype, a descriptive synopsis of opioid-related data will be compiled, including conditions or variables associated with opioid or µOR activity influencing malignancy or its treatment. The analysis will be split into 2 main components based on the “Research question.”

#### Component 1: Do Therapeutically Relevant Concentrations of Opioids Affect Tumor Growth and Survival?

Data extracted from clinical and basic research studies will be aggregated by µOR ligand and cancer type. µOR ligands will be categorized by class (agonist, antagonist, and partial agonist) as well as by individual entity (morphine, oxycodone, etc) and by measures of µOR ligand exposure (eg, concentrations, PK parameter estimates, dosage, and route of administration) or exposure-response (eg, IC_50_).

For basic (animal, in vitro) study results, µOR ligands will be categorized according to effect on malignancy (ie, no effect, promotes, or inhibits malignancy), and for clinical study results, µOR ligands will be categorized according to effect on malignancy, chemotherapeutic activity via clinical indices, or patient outcome (eg, minimal residual disease and progression-free survival). Clinical data will be further categorized by covariates such as sex, age range (pediatric vs adult), new diagnosis, or relapse.

Due to differences in study design, direct comparisons between studies are not feasible. Data will be aggregated by category, and a count of results will be conducted. An illustration: consider a study that demonstrates that morphine administered in high doses, generating supraphysiologic blood concentrations (considered toxic for humans), stimulated metastases, as compared to controls, in a xenograft murine model of breast cancer. This study result would be tabulated as breast cancer, µOR agonist, supraphysiologic exposure, promotes cancer. If, in addition, to the experimental conditions above, morphine was administered to generate clinically relevant blood concentrations, and these were associated with diminished tumor growth, this would be tabulated as breast cancer, µOR agonist, physiologic exposure, inhibits cancer.

#### Component 2: Do Therapeutically Relevant Concentrations of Opioids Alter the Antineoplastic Activity of Chemotherapeutics?

Data extracted from clinical and basic research (preclinical) studies, incorporating µOR ligand use coinciding with chemotherapeutics used to induce malignant cell death will be aggregated by µOR ligand, cancer type, and chemotherapeutic class.

Chemotherapeutics and µOR ligands will be classified by class as well as by individual entity. Chemotherapeutics will include conventional (anthracyclines, antimetabolites, alkylating agents, mitotic spindle inhibitors, topoisomerase inhibitors, antitumor antibiotics, platinum-based agents, corticosteroids, biologicals [enzymes], or nitrosoureas), targeted (kinase inhibitors, proteasome inhibitors, and antibodies), and agents undergoing clinical trials or considered experimental.

Measures of chemotherapeutic agent or µOR ligand exposure (eg, concentrations, PK parameter estimates, dosage, and route of administration) or exposure-response (eg, IC_50_) will be considered.

For basic (animal, in vitro) study results, µOR ligands will be categorized according to effect on chemotherapeutic activity (ie, no effect, antagonism, or synergism). For clinical study results, µOR ligands will be categorized according to the effect on chemotherapeutic activity via clinical indices and patient outcomes studies attribute to or suggest as being related to changes in treatment efficacy. Clinical data will be further categorized by covariates such as sex, age range (pediatric vs adult), new diagnosis, or relapse.

As with component 1, it is anticipated that differences in study design and experimental conditions will preclude direct comparisons between data generated across studies. As above, data will be tabulated by category, including chemotherapy type, and a count of results will be conducted.

### Ethical Considerations

This study does not require ethical approval as data and information collected, using scoping review methodology, has previously been made available through publication. The results of this scoping review will be submitted for publication in a relevant peer-reviewed journal as well as disseminated through presentations at scientific conferences. Any changes from our protocol during the conduct of the scoping review will be acknowledged and defined in the manuscript.

## Results

The results of this scoping review will be presented using a descriptive narrative form as well as graphical and tabular representations. The results will be structured, as appropriate, to emphasize the effects of prescription opioids by cancer classification as well as chemotherapy type. Additionally, study results concerning data classified as preclinical, translational, and clinical will be presented distinctly to facilitate comparison. The protocol for the scoping review was initiated in February 2021 at the University of Utah and is expected to be completed in August 2023. The results of the scoping review will be disseminated through regional and national scientific conference proceedings and presentations, stakeholder meetings, and by publication in a peer-reviewed journal.

## Discussion

This scoping review will map diverse studies spanning from preclinical to clinical regarding the intersection of opioids with malignancy and its treatment. The interpretation of opioid-malignancy interactions within a clinical context is often confounded by pain and its management. It is therefore both novel and necessary to include preclinical study data to identify key knowledge gaps, begin to reconcile seeming conflicting data (eg, opioids foment cancer growth vs opioids kill cancer cells) by setting a context to parse the biologic and molecular complexities of opioid impact on malignancy and its treatment, and ultimately, inform patient treatment algorithms to optimize outcomes. The knowledge presented in the scoping review will help to inform and improve the design of studies to assess the unmet medical need to understand the risks and benefits associated with opioids extending to the potential to impact cancer and its care.

This scoping review study has several strengths. First, our scoping review approach will be inclusive of studies spanning the spectrum of published data available from clinical to in vitro. Popular opinion and clinical judgment regarding the potential for opioids to affect malignancy have been deeply influenced by the publication of basic (in vitro or ex vivo) studies. Therefore, expanding this review to include both clinical and preclinical studies provides a new perspective on the topic, acknowledges that basic studies impact clinical care and shape patients’ attitudes, and addresses limitations specific to the opioid-malignancy question in the context of clinical studies. Current clinical studies examining the opioid-malignancy question, often retrospective, are confounded by pain and the management of pain. Adequate pain management is associated with improved patient survival; therefore, interpretation of the potential effects of opioid use on the malignancy itself is challenging to interpret. Finally, despite the intense attention the topic has received, the key dimension of opioid concentration is often not addressed. Therefore, incorporating data from clinical and preclinical studies into this review with an emphasis on systemic opioid exposures (concentration) is warranted, and based on the preliminary data collected, aids in clarifying seemingly contradictory findings pointed to in existing reviews.

This scoping review also has limitations. Specifically, no quality assessment of the included studies will be performed. Relevant data may be missed, as we are searching for published studies only. We selected several sources and developed sensitive search strategies to increase the discovery of eligible studies.

The topic of opioid impact on cancer and its treatment is not new but remains controversial and clinically relevant. This scoping review seeks to encompass preclinical and clinical studies to generate a comprehensive landscape for interpreting the existing literature within pathophysiologic and pharmacologic contexts. We hope this study will be used to identify key knowledge gaps and advance future studies aimed at improving patient outcomes.

## Appendix

Multimedia Appendix 1 Peer review reports.

## Abbreviations

µOR: µ-opioid receptor
PK: pharmacokinetic
PRISMA-ScR: Preferred Reporting Items for Systematic Reviews and Meta-Analyses extension for Scoping Reviews
REDCap: Research Electronic Data Capture

## Data Availability

Data sets will be presented in the main manuscript and as additional supporting files or made available upon request.
