# Supplementary material for: Mapping the Evidence for Opioid-Mediated Changes in Malignancy and Chemotherapeutic Efficacy: Protocol for a Scoping Review
Source: JMIR Res Protoc. 2023 May 22;12:e38167. doi: 10.2196/38167 (PMC10242459; doi:10.2196/38167)
Supplement: Multimedia Appendix 1 [file resprot_v12i1e38167_app1.pdf]

SUMMARY STATEMENT
(Privileged Communication)

PROGRAM CONTACT:
SONIA JAKOWLEW
240-276-5630
jakowles@mail.nih.gov

Release Date: 05/21/2021
Revised Date:

Application Number: 1 K22 CA258671-01A1
Formerly: 1K22CA258671-01

Principal Investigator

CONSTANCE, JONATHAN ERIC

Applicant Organization: UNIVERSITY OF UTAH

Review Group: ZCA1 RTRB-R (O2)
National Cancer Institute Special Emphasis Panel
K22 Transition Career Development Award

Meeting Date: 05/18/2021
Council: OCT 2021
Requested Start: 09/01/2021
RFA/PA: PAR18-467
PCC: 1STR

Project Title: Opioid-induced changes to chemotherapeutic activity in blood cancer

SRG Action: Impact Score:16
Next Steps: Visit https://grants.nih.gov/grants/next_steps.htm
Human Subjects: 30-Human subjects involved - Certified, no SRG concerns
Animal Subjects: 10-No live vertebrate animals involved for competing appl.
Gender: 1A-Both genders, scientifically acceptable
Minority: 1A-Minorities and non-minorities, scientifically acceptable
Age: 6A-Children and Adults, scientifically acceptable

Project Year	Direct Costs Requested	Estimated Total Cost
1	183,656	198,348
2	186,534	201,457
3	186,235	201,134
TOTAL	556,425	600,939

ADMINISTRATIVE BUDGET NOTE: The budget shown is the requested budget and has not been adjusted to reflect any recommendations made by reviewers. If an award is planned, the costs will be calculated by Institute grants management staff based on the recommendations outlined below in the COMMITTEE BUDGET RECOMMENDATIONS section.

RESUME AND SUMMARY OF DISCUSSION: The applicant proposes to determine the impact of opioid-chemotherapy interactions on chemotherapeutic efficacy in blood cancers in children, adolescents, and young adults (CAYAs). The hypothesis is that chemotherapeutic response will change in the presence of clinically-relevant concentrations of opioids that are potent activators of the mu-opioid receptor (μOR; OPRM1 gene), expressed in many cancer types. This is a resubmitted application and the applicant has adequately-responded to most of the criticisms in the previous review. The applicant has already made significant progress and provides preliminary data showing opioids-induced resistance to molecularly-targeted chemotherapy, with an impact dependent on the cancer type. He has 15 first-author papers from his post-doctoral training and more than 30 total published papers, including senior-author ones. The letters of recommendation are each very strong and describe the applicant as highly-qualified, productive, and very knowledgeable in the field of pharmacology. He was accepted to the American College of Clinical Pharmacology Inaugural Leadership Development Program, received the Center for Clinical and Translational Science and Primary Children's Hospital Foundation Career Development Award, and was selected as the Vice President's Clinical and Translational Research Scholar. These awards attest to his trajectory to be a successful and independent investigator. The research plan aligns well with the candidate's training and the plan to become a competent translational scientist in clinical and survivorship care. If successful, this research plan will likely lead to strong publications. The career development plan/career goals and objectives are relevant to the proposed work. The applicant has assembled a strong team of consultants/collaborators. The research environment at the University of Utah School of Medicine is well-suited for the proposed research and there is institutional commitment to the candidate to support a transition to an independent faculty position at another institution. The applicant will learn many new techniques and he provides a training plan that is consistent with his goal of becoming a cancer researcher. The strong candidate, the carefully-considered career development plan, and the skill development that will result from the proposed research and training mitigate the minor weaknesses in the design of the research plan regarding the many levels of variability present in this study: type of leukemia in human specimens, type of cell line, level of opioids in blood samples of CAYAs, whether the opioids augment or inhibit malignancy, repeated measures, etc. Nevertheless, overall, it is likely that the activities described in this application, will provide strong training to advance the applicant's research independence to a tenured faculty position.

DESCRIPTION (provided by applicant): Routinely prescribed analgesic opioids are potent activators of the mu-opioid receptor (μOR; OPRM1 gene), expressed in many cancer types, and can impact cancer cell survival and the efficacy of lifesaving chemotherapy. For patients with cancer, opioid use often coincides with chemotherapy, making opioid- chemotherapy interactions inevitable. For some cancers, including lung, prostate, gastric, breast, and esophageal cancers, opioid use and increased μOR expression are linked to increased tumor growth, metastases, and shorter patient survival. In contrast, in vitro and animal model studies for glioblastoma, certain breast cancers, and T- and B-cell acute leukemias, opioids stimulate cancer cell death and, in some cases, enhance cytotoxic chemotherapeutic response. The seemingly paradoxical effects likely have a concentration- dependent dimension as physiologic opioid exposures have tended to induce pro-proliferative effects while supraphysiologic opioid exposures are typically associated with cancer cell death. While μOR activation can enhance the killing effect of genotoxic chemotherapy in acute lymphoblastic leukemia, our preliminary data demonstrate opioids antagonize the apoptotic response of Philadelphia chromosome-positive (Ph+) chronic myeloid leukemia cells (K562) to molecularly-targeted tyrosine kinase inhibitor (TKI) chemotherapy. As leukemias are treated with both genotoxic and molecularly targeted chemotherapy assessing the potential for clinically used opioids to antagonize or synergize in leukemic cell killing is an urgent medical need. We propose to test the central hypothesis, that chemotherapeutic response will change in the presence of clinically relevant concentrations of opioids, in three Aims. Specific Aim 1: Quantify standard-of-care opioid exposures and determinants of exposure in patients

with leukemia. Hypothesis 1: Interpatient variability in opioid exposure will exceed 50% due to inherent metabolic differences, disease status, and treatment- related pharmacokinetic alterations. Specific Aim 2: In leukemic cell lines, changes in response to chemotherapy based on leukemic subtype and µOR function will be determined. Hypothesis 2: Clinically- experienced concentrations of opioids will change chemotherapeutic response in different leukemic subtypes corresponding with µOR function by >25%. Specific Aim 3: In patients with leukemia, the frequency of opioid- chemotherapy DDIs based on clinical and molecular factors will be determined. Hypothesis 3: Clinical and molecular features associated with opioid-chemotherapy DDI conferring chemotherapy resistance are present in >20% of patients prescribed opioids. Understanding the impact of µOR activity on chemotherapeutic response across similar but biologically distinct leukemia cell types will provide new insights into mechanisms underlying drug resistance, relapse, or non-response and drive precision medicine in opioid prescribing. This application will provide key preliminary data to support an NIH R01 aimed at predicting altered chemotherapeutic response due to supportive care medication exposure among patients undergoing treatment for leukemia.

PUBLIC HEALTH RELEVANCE: It is desirable to avoid detrimental drug interactions to ensure each patient achieves the best possible response to their chemotherapy. The goal of this research application is to determine the impact of opioid- chemotherapy interactions on chemotherapeutic efficacy in blood cancer.

CRITIQUE: The written critiques of individual reviewers are provided in essentially unedited form in this section. Please note that critiques and criteria scores, prepared prior to the review meeting, may not have been revised following discussions at the meeting. The "Resume and Summary of Discussion" section summarizes the final opinions of the review committee.

CRITIQUE 1

Candidate:	1
Career Development Plan/Career Goals /Plan to Provide Mentoring:	3
Research Plan:	2
Mentor(s), Co-Mentor(s), Consultant(s), Collaborator(s):	1
Environment Commitment to the Candidate:	1

Overall Impact

This re-submission comes from a highly-qualified candidate, who is currently a research assistant professor in the Department of Pediatrics at the University of Utah School of Medicine who has a focus on pediatric oncology research. The candidate is productive, has institutional support for an independent research career and has an excellent career development plan with a Career Advisory Team. Although no major weaknesses were noted, some limitations include the need for more epidemiology and applied biostatistics in the research plan and a clear plan for how the current study that embraces variability will provide key evidence for the next R01.

1. Candidate

Strengths

- High quality training: PhD in pharmacology and toxicology (published 7 articles from dissertation), training in molecular biology, and board certified in clinical pharmacology.
- Productive throughout PhD training and post-doctoral training (>30 publications) and runs a translational research program since 2015 supported in part by internal institution funding.

- Seeks to get advanced training in phospho-flow cytometry, pharmacometrics, the conduct of pharmaco-epidemiologic studies requiring very large data sets, and translational research in the setting of clinical management and care of patients with pediatric malignancies. The latter two training goals are very relevant and appropriate for translating his research to human populations, specifically in children, adolescents, and young adults (CAYA).
- Letters are strongly supportive of his research goals and indicate a willingness of the writers to continue to collaborate and work with Dr. Constance. The letter from the Chair of Pediatrics indicates a high confidence in an independent research career for Dr. Constance.

Weaknesses

- None noted.

2. Career Development Plan/Career Goals & Objectives

Strengths

- With one exception (see below), the scientific training goals, the composition of the Career Advisor Team, the timeline (3 years), and the career development activities (Research Investigator Certificate, Responsible Conduct of Research, Grantsmanship, External Advisors, Professional Societies, Huntsman Cancer Institute membership, Leadership) are all appropriate career goals.
- The candidate has built the Career Advisory Team (Drs. Deininger, Lemons, Lim, and Watt), whom he will meet with on a quarterly basis to discuss his research and career development progress as well as progress in achieving independent R01 research grant funding.
- The timeline presented in Table 2 is very reasonable with the first milestone (Aim 1) to be achieved at the end of the second quarter in year 2.

Weaknesses

- The candidate notes that the Greater Intermountain Node (GIN) Database Opioid Research Consultation (DORC) group monthly meetings will advance his knowledge of pharmaco-epidemiology. Additionally, the candidate proposes to use the Intermountain Data Services for observational studies (see budget). There are extensive resources and expertise at UT, especially at the Huntsman, for epidemiology, pharmaco-epidemiology, study design, analysis and population science in general. If the candidate is serious about becoming a competent translational scientist in clinical and survivorship care, this seems like a missed opportunity. The candidate is encouraged to seek out these opportunities and capitalize on them.
- (minor) The timeline presented in Table 2 has Aim 3 extending all the way to the end of year 3 (and beyond?). However, data from this aim may be critical for an R01 research grant application. What are the milestones/data acquisition and analysis that need to be completed before the end of year 3 for a robust R01 research grant application?

3. Research Plan

Strengths

- Drug-drug interactions (DDIs) are generally understudied in cancer treatment and the lack of such knowledge makes it difficult to optimize dosing of the drugs.
- The specific DDI of opioids and chemotherapy are relevant for CAYAs, but probably also for many adult cancers treated with chemotherapy (e.g., ovarian cancer), leading to a broader application of this work if successful.

- The availability of the biorepository of the scavenge blood samples for CAYA leukemia patients is a great asset to the project.
- Specific aims are clear and appear to be doable in the environment.

Weaknesses

- Variability can be helpful and hurtful in such a study. There are many level of variability present in this study: type of leukemia in human specimens; type of cell line; level of opioids in blood samples of CAYAs; whether the opioids augment or inhibit malignancy; repeated measures (Aim 3); etc. There can also be positive or negative correlations between all these sources of variability. While the candidate is correct in saying that this represents “real-world variability”, it will also make it difficult get actionable next steps from the data/results that will translatable to the next step of research.

4. Consultant(s), Collaborator(s)

Strengths

- The candidate has established outstanding collaborators, who have relevant expertise and the Career Advisory Team that can help him with his research, as well as his career development.

Weaknesses

- An applied biostatistician or pharmaco-epidemiologist may enhance the project and the candidate training.

5. Environment and Institutional Commitment to the Candidate

Strengths

- Access to a critical biorepository.
- Strong letter of support from the Department Chair of Pediatrics.
- Highly-supportive environment, as noted by the letters of collaboration and availability of services.

Weaknesses

- None noted.

ADDITIONAL REVIEW CRITERIA

Protections for Human Subjects

Acceptable Risks and Adequate Protections

- Human Subjects protections are in line with samples from a biorepository that fall under a waiver of consent.

Data and Safety Monitoring Plan (Applicable for Clinical Trials Only):

Not Applicable (No Clinical Trials)

Inclusion Plans

- Sex/Gender: Distribution justified scientifically
- Race/Ethnicity: Distribution justified scientifically
- For NIH-Defined Phase III trials, Plans for valid design and analysis: Not Applicable
- Inclusion/Exclusion Based on Age: Distribution justified scientifically

Vertebrate Animals

Not Applicable (No Vertebrate Animals)

Biohazards

Not Applicable (No Biohazards)

Resubmission

- Generally a responsive re-submission.

ADDITIONAL REVIEW CONSIDERATIONS

Training in the Responsible Conduct of Research

Acceptable

Comments on Format (Required):

- IRB training; RCR course- part of a larger Research Investigator Certificate (RATS CRC course).

Comments on Subject Matter (Required):

- Not specified but all courses were noted to meet University and NIH criteria.

Comments on Faculty Participation (Required; not applicable for mid- and senior-career awards):

- On-going, informal training in RCR.

Comments on Duration (Required):

- IRB training -online (unknown duration); RATS RCR course- part of a larger Research Investigator Certificate (unknown duration);

Comments on Frequency (Required):

- IRB training -online (every two years); RATS CRC course- part of a larger Research Investigator Certificate (every 4 years)

Resource Sharing Plans

Acceptable

Authentication of Key Biological and/or Chemical Resources

Not Applicable (No Relevant Resources)

Budget and Period of Support

Recommend as Requested

CRITIQUE 2

Candidate:	1
Career Development Plan/Career Goals /Plan to Provide Mentoring:	2
Research Plan:	2
Mentor(s), Co-Mentor(s), Consultant(s), Collaborator(s):	1
Environment Commitment to the Candidate:	2

Overall Impact

This applicant addresses an important topic, namely the interaction between opioids and chemotherapy effectiveness. The focus is on childhood, adolescent, and young adult (CAYA) cancer patients, for whom the frequency of opioid treatment is high. The studies leverage the professional strengths of the candidate and include population pharmacokinetics, mechanistic studies of drug-drug-interactions and interrogation of patient specimens for early translation. The training plan and research aims are well-integrated. This is a very strong application, with likely clinically impactful results.

1. Candidate

Strengths

- The candidate earned a bachelor degree in chemistry, followed by a Ph.D. in pharmacology/toxicology, and is a board accredited clinical pharmacologist.
- The applicant has both first- and senior-author publications studying opioids in cancer patients, and has a substantial record of first authored papers focused on leukemia chemotherapy and polypharmacy. Expertise in pediatric cancers has also been demonstrated.
- The highly productive postdoctoral period yielded 15 first authored papers and numerous collaborative works, indicative of the strong interdisciplinary environment.
- The applicant has received institutional investments, including 90% release for research and a KL2-type institutional training award.

Weaknesses

- None noted.

2. Career Development Plan/Career Goals & Objectives

Strengths

- The candidate will be committed 90% to research, and has identified a strong plan that includes training, mentorship, research, and professional development activities.
- A research scholarship program for external mentorship complements an outstanding mentoring team within the organization.
- Course-work in genomics, a focus of the research plan, is included.
- Leadership training plans are outlined, including a principal investigator certification plan.
- Formal annual evaluation is outlined, as well as a plan for transitioning to independent funding.
- A specific, measurable plan for manuscripts and a detailed timeline are included.
- RCR training is addressed well.

Weaknesses

- None noted.

3. Research Plan

Strengths

- The applicant is responsive to previous concerns about the research plan.
- The applicant documents the prevalence of opioid use, making a strong case for the importance of population PK measures to establish physiologically-relevant doses.
- The aims are independent, because physiologic ranges can be approximated from early in the study.
- In a cell-based study, ~10 cell lines with various traits, all derived from leukemia, will be tested for chemosensitivity with and without opioid exposure, considering mu-opioid receptor expression as a mediator. High-throughput screening assays will be employed, which have ancillary training benefits.
- The research aims are well-integrated with training aims.

Weaknesses

- None noted.

4. Consultant(s), Collaborator(s)

Strengths

- The consultants and collaborators include a hematologic oncologist, a pediatric oncologist, a cancer biologist, and a pharmacologist, all of whom provide meaningful letters of support. The expertise is complementary and an annual plan for formal evaluation by this group is built into the training plan.

Weaknesses

- None noted.

5. Environment and Institutional Commitment to the Candidate

Strengths

- The institution is strong and well-suited to the applicant’s research focus area.
- The availability of strong interdisciplinary collaborators relevant to the project speaks to the institutions strength in the area of research.
- The institutions has demonstrated investment, both in release time and in the awarding of the KL2-like training mechanism.

Weaknesses

- None noted.

ADDITIONAL REVIEW CRITERIA

Protections for Human Subjects

Acceptable Risks and Adequate Protections

Data and Safety Monitoring Plan (Applicable for Clinical Trials Only):
Not Applicable (No Clinical Trials)

Inclusion Plans

- Sex/Gender: Distribution justified scientifically
- Race/Ethnicity: Distribution justified scientifically
- For NIH-Defined Phase III trials, Plans for valid design and analysis: Not applicable
- Inclusion/Exclusion Based on Age: Distribution justified scientifically

Vertebrate Animals
NO, animal welfare concerns or incomplete

Biohazards
Not Applicable (No Biohazards)

Resubmission

- Very responsive to previous concerns.

ADDITIONAL REVIEW CONSIDERATIONS

Training in the Responsible Conduct of Research
Acceptable
Comments on Format (Required):

- Formal course-work is included.

Comments on Subject Matter (Required):

- All relevant content areas are included.

Comments on Faculty Participation (Required; not applicable for mid- and senior-career awards):

- The applicant has detailed plans for participation.

Comments on Duration (Required):

- Throughout training award.

Comments on Frequency (Required):

- Sufficient frequency.

Resource Sharing Plans
Not Applicable (No Relevant Resources)

Authentication of Key Biological and/or Chemical Resources

Not Applicable (No Relevant Resources)

Budget and Period of Support

Recommend as Requested

CRITIQUE 3

Candidate:	2
Career Development Plan/Career Goals /Plan to Provide Mentoring:	1
Research Plan:	2
Mentor(s), Co-Mentor(s), Consultant(s), Collaborator(s):	1
Environment Commitment to the Candidate:	1

Overall Impact

This is a resubmission of a K22 career development grant from Dr. Jonathan Constance, who is a board accredited clinical pharmacologist, who proposes to address and important clinical research question using multiple analytical methods to understand detrimental opioid chemotherapy interactions on chemotherapeutic efficacy in blood cancer in children. The proposed research is important to determine the best possible response to chemotherapy. The application is well-written and highly-responsive to the prior critique. Since the submission, he received a Primary Children's Hospital Foundation Career Development grant. He has also provided preliminary data for this research showing the opioids-induced resistance to molecularly-targeted chemotherapy, but the impact is dependent on the cancer type. This is an important question, and it what is learned from this study could be important for adult-onset cancers. His research methods are novel and the clinical translation is important. His letters are very strong, and attest to his knowledge of the field and his trajectory as an independent researcher.

1. Candidate

Strengths

- Dr. Constance has received outstanding training as a pharmacologist, and completed a post-doctoral fellowship in clinical pharmacology (board accredited) and completed a fellowship in pediatric clinical pharmacology (relevant to this application).
- He was accepted to the American College of Clinical Pharmacology Inaugural Leadership Development Program, received a Center for Clinical and Translational Science and Primary Children's Hospital Foundation Career Development Award, and was selected as the Vice President's Clinical and Translational Research Scholar. These awards attest to his trajectory to be a successful and independent investigator.
- Candidates letters are outstanding.

Weaknesses

- It was mentioned in the prior review that the candidate lacked publications (first-author) on the topic of this application, and this was not addressed in the introduction. It would have been good to see an explanation. He does have 2 publications on the topic but in one (Leukemia and Lymphoma) he is last author (not senior), and the other published in Clinical Translational Science he is a middle author.

- He was co-author on the 2 papers (2020 and 2021) and nothing between 2020 and 2017. However, the papers in 2017 he is first-author and they are relevant to the topic of this application.

2. Career Development Plan/Career Goals & Objectives

Strengths

- Dr. Constance has a clear training and career path outlined. He proposes to include training in advanced methods in phospho-flow cytometry, pharmacometrics, pharmaco-epidemiologic methods using large data sets, and translational research in the setting of clinical management and care of patients with pediatric malignancies. These skills will be important for his transition to independence.
- He also proposes to participate in other work-shops and leadership programs.
- His mentors are all willing and available to continue mentoring.

Weaknesses

- None noted.

3. Research Plan

Strengths

- The candidate proposes three specific aims to test his central hypothesis that chemotherapy response will change with clinically-relevant concentrations of opioids. This research question is important and significant for improving patient outcomes.
- The concerns about clinical translation has been addressed.

Weaknesses

- None noted.

4. Consultant(s), Collaborator(s)

Strengths

- Dr. Constance assembled 4 career advisors in complementary content areas (oncology, pediatric oncology, cancer biology, and pharmacology). All are supportive to work with Dr. Constance and the letters are excellent in showing their support.
- He uses multiple approaches to test this hypothesis, including use of cell lines.

Weaknesses

- None noted.

5. Environment and Institutional Commitment to the Candidate

Strengths

- There are no concerns regarding the commitment of University of Utah to support the candidate and the environment is excellent for Dr. Constance to complete the work proposed.
- Dr. Giardino, Chair of Pediatrics at the University of Utah writes a very strong support letter, confirming 75% protected research time to conduct the research in this K22 career development award.

Weaknesses

- None noted.

ADDITIONAL REVIEW CRITERIA

Protections for Human Subjects

Acceptable Risks and Adequate Protections

Data and Safety Monitoring Plan (Applicable for Clinical Trials Only):

Not Applicable (No Clinical Trials)

Inclusion Plans

- Sex/Gender: Distribution justified scientifically
- Race/Ethnicity: Distribution justified scientifically
- For NIH-Defined Phase III trials, Plans for valid design and analysis: Not applicable
- Inclusion/Exclusion Based on Age: Distribution justified scientifically

Vertebrate Animals

Not Applicable (No Vertebrate Animals)

Biohazards

Not Applicable (No Biohazards)

Resubmission

- Responsive to the previous review critiques.

ADDITIONAL REVIEW CONSIDERATIONS

Training in the Responsible Conduct of Research

Acceptable

Comments on Format (Required):

- lectures, group discussions, and a comprehensive online class with a proficiency examination.

Comments on Subject Matter (Required):

- Acceptable

Comments on Faculty Participation (Required; not applicable for mid- and senior-career awards):

- Taught by University Faculty and staff with experience on subject matter.

Comments on Duration (Required):

- 10 hours.

Comments on Frequency (Required):

- Repeat every 4 years.

Resource Sharing Plans

Acceptable

Authentication of Key Biological and/or Chemical Resources

Not Applicable (No Relevant Resources)

Budget and Period of Support

Recommend as Requested

THE FOLLOWING SECTIONS WERE PREPARED BY THE SCIENTIFIC REVIEW OFFICER TO SUMMARIZE THE OUTCOME OF DISCUSSIONS OF THE REVIEW COMMITTEE, OR REVIEWERS' WRITTEN CRITIQUES, ON THE FOLLOWING ISSUES:

PROTECTION OF HUMAN SUBJECTS: ACCEPTABLE
The study will involve secondary research with deidentified biospecimen from pediatric B-ALL biopsies.

INCLUSION OF WOMEN PLAN: ACCEPTABLE G1A
Girls will be included.

INCLUSION OF MINORITIES PLAN: ACCEPTABLE M1A
Minorities will be included.

INCLUSION ACROSS THE LIFESPAN: ACCEPTABLE C6A
Children and young adults in the age range of 1-26 will be included.

COMMITTEE BUDGET RECOMMENDATIONS: The budget was recommended as requested.

Footnotes for 1 K22 CA258671-01A1; PI Name: CONSTANCE, JONATHAN ERIC

NIH has modified its policy regarding the receipt of resubmissions (amended applications).See Guide Notice NOT-OD-18-197 at https://grants.nih.gov/grants/guide/notice-files/NOT-OD-18-197.html. The impact/priority score is calculated after discussion of an application by averaging the overall scores (1-9) given by all voting reviewers on the committee and multiplying by 10. The criterion scores are submitted prior to the meeting by the individual reviewers assigned to an application, and are not discussed specifically at the review meeting or calculated into the overall impact score. Some applications also receive a percentile ranking. For details on the review process, see http://grants.nih.gov/grants/peer_review_process.htm#scoring.

MEETING ROSTER

National Cancer Institute Special Emphasis Panel
NATIONAL CANCER INSTITUTE
K22 Transition Career Development Award

ZCA1 RTRB-R (O2)
05/18/2021

Notice of NIH Policy to All Applicants: Meeting rosters are provided for information purposes only. Applicant investigators and institutional officials must not communicate directly with study section members about an application before or after the review. Failure to observe this policy will create a serious breach of integrity in the peer review process, and may lead to actions outlined in NOT-OD-14-073 at https://grants.nih.gov/grants/guide/notice-files/NOT-OD-14-073.html and NOT-OD-15-106 at https://grants.nih.gov/grants/guide/notice-files/NOT-OD-15-106.html, including removal of the application from immediate review.

CHAIRPERSON(S)

TEW, KENNETH D., PHD, DSC
PROFESSOR, CHAIR
DEPARTMENT OF CELL AND MOLECULAR PHARMACOLOGY
AND EXPERIMENTAL THERAPEUTICS
HOLLINGS CANCER CENTER
MEDICAL UNIVERSITY OF SOUTH CAROLINA
CHARLESTON, SC 29425

MEMBERS

AMBULOS, NICHOLAS P JR, PHD
ASSOCIATE PROFESSOR AND DIRECTOR
BIOPOLYMER CORE FACILITY
DEPARTMENT OF MICROBIOLOGY AND IMMUNOLOGY
SCHOOL OF MEDICINE
UNIVERSITY OF MARYLAND
BALTIMORE, MD 21201

APLIN, ANDREW E, PHD
PROFESSOR
ASSOCIATE DIRECTOR, BASIC SCIENCES
DEPARTMENT OF CANCER BIOLOGY
SIDNEY KIMMEL CANCER CENTER
THOMAS JEFFERSON UNIVERSITY
PHILADELPHIA, PA 19107

ASHENDEL, CURTIS L., PHD
ASSOCIATE PROFESSOR
DEPARTMENT OF MEDICINAL CHEMISTRY AND MOLECULAR
PHARMACOLOGY
PURDUE UNIVERSITY
WEST LAFAYETTE, IN 47907

BARTLETT, DAVID BRUCE, PHD
ASSISTANT PROFESSOR
DEPARTMENT OF MEDICINE
DIVISION OF MEDICAL ONCOLOGY
DUKE MOLECULAR PHYSIOLOGY INSTITUTE
DUKE UNIVERSITY SCHOOL OF MEDICINE
DURHAM, NC 27701

BLACK, JENNIFER D., PHD
PROFESSOR; LEADER, GASTROINTESTINAL CANCER
PROGRAM
EPPLEY INSTITUTE FOR RESEARCH IN CANCER AND ALLIED
FRED AND PAMELA BUFFETT CANCER CENTER
UNIVERSITY OF NEBRASKA MEDICAL CENTER
OMAHA, NE 68198

BONDY, MELISSA L., PHD
CHAIR AND PROFESSOR
DEPARTMENT OF EPIDEMIOLOGY AND POPULATION
HEALTH
ASSOCIATE DIRECTOR FOR POPULATION SCIENCE
CENTER FOR POPULATION HEALTH SCIENCES
STANFORD CANCER INSTITUTE; STANFORD UNIVERSITY
STANFORD, CA 94305

BUCHSBAUM, DONALD J., PHD
PROFESSOR AND DIRECTOR
DIVISION OF RADIATION BIOLOGY
DEPARTMENT OF RADIATION ONCOLOGY
UNIVERSITY OF ALABAMA AT BIRMINGHAM
BIRMINGHAM, AL 35294-3300

COLLER, HILARY A, PHD
PROFESSOR
DEPARTMENT OF MOLECULAR, CELL
AND DEVELOPMENTAL BIOLOGY
UNIVERSITY OF CALIFORNIA, LOS ANGELES
LOS ANGELES, CA 90095

COOK, LINDA S, PHD
PROFESSOR
DEPARTMENT OF INTERNAL MEDICINE
DIVISION OF EPIDEMIOLOGY, BIOSTATISTICS,
AND PREVENTIVE MEDICINE
UNIVERSITY OF NEW MEXICO
ALBUQUERQUE, NM 87131

DE GROOT, JOHN F, MD
PROFESSOR INTERIM CHAIR
DEPARTMENT OF NEURO-ONCOLOGY
UNIVERSITY OF TEXAS
MD ANDERSON CANCER CENTER
HOUSTON, TX 77030

DONOGHUE, DANIEL J, PHD
PROFESSOR, PROVOST SIXTH COLLEGE
DEPARTMENTS OF CHEMISTRY AND BIOCHEMISTRY
MOORES CANCER CENTER
UNIVERSITY OF CALIFORNIA, SAN DIEGO
LA JOLLA, CA 92093-0367

FIELDS, TIMOTHY A, MD, PHD
DIRECTOR MEDICAL SCIENTISTS TRAINING PROGRAM, PROFESSOR
DEPARTMENT OF PATHOLOGY AND LABORATORY MEDICINE
UNIVERSITY OF KANSAS
KANSAS CITY, KS 66160

GMEINER, WILLIAM H, PHD
PROFESSOR
DEPARTMENT OF CANCER BIOLOGY, PHYSIOLOGY, AND PHARMACOLOGY; SCHOOL OF MEDICINE
BAPTIST COMPREHENSIVE CANCER CENTER
WAKE FOREST UNIVERSITY
WINSTON-SALEM, NC 27157

JIANG, YU, PHD
PROFESSOR
DEPARTMENT OF PHARMACOLOGY AND CHEMICAL BIOLOGY
HILLMAN CANCER CENTER
UNIVERSITY OF PITTSBURGH
PITTSBURGH, PA 15261

KARLSEDER, JAN, PHD
PROFESSOR, CHAIR
DEPARTMENT OF MOLECULAR AND CELLULAR BIOLOGY
THE SALK INSTITUTE
LA JOLLA, CA 92037

KERR, WILLIAM GARROW, PHD
PROFESSOR
DEPARTMENT OF PEDIATRICS AND MICROBIOLOGY AND IMMUNOLOGY
STATE UNIVERSITY OF NEW YORK
SYRACUSE, NY 13210

SHENG, SHIJIE, PHD
PROFESSOR
DEPARTMENT OF PATHOLOGY AND ONCOLOGY
KARMANOS CANCER INSTITUTE
WAYNE STATE UNIVERSITY SCHOOL OF MEDICINE
DETROIT, MI 48201

SPITZ, DOUGLAS ROBERT JR, PHD
PROFESSOR AND DIRECTOR
DEPARTMENT OF RADIATION ONCOLOGY
FREE RADICAL AND RADIATION BIOLOGY PROGRAM
HOLDEN COMPREHENSIVE CANCER CENTER
UNIVERSITY OF IOWA
IOWA CITY, IA 52242

TROESTER, MELISSA A., PHD, MPH
PROFESSOR
DEPARTMENT OF EPIDEMIOLOGY
LINEBERGER CANCER CENTER
UNIVERSITY OF NORTH CAROLINA AT CHAPEL HILL
CHAPEL HILL, NC 27599

ZHANG, RUIWEN, MD, PHD
PROFESSOR
ROBERT L. BOBLITT ENDOWED CHAIR IN DRUG DISCOVERY
DIRECTOR, CENTER FOR DRUG DISCOVERY
DEPARTMENT OF PHARMACOLOGY AND TOXICOLOGY
UNIVERSITY OF HOUSTON COLLEGE OF PHARMACY
HOUSTON, TX 77204

SCIENTIFIC REVIEW OFFICER

STOICA, ADRIANA, PHD
SCIENTIFIC REVIEW OFFICER
RESOURCES AND TRAINING REVIEW BRANCH
DIVISION OF EXTRAMURAL ACTIVITIES
NATIONAL CANCER INSTITUTE
NATIONAL INSTITUTES OF HEALTH
ROCKVILLE, MD 20850

EXTRAMURAL SUPPORT ASSISTANT

WILSON, BRIDGETTE
EXTRAMURAL SUPPORT ASSISTANT
RESOURCE & TRAINING REVIEW BRANCH
DIVISION OF EXTRAMURAL ACTIVITIES
NATIONAL CANCER INSTITUTE, NIH
NATIONAL INSTITUTES OF HEALTH
BETHESDA, MD 20892

PROGRAM REPRESENTATIVE

JAKOWLEW, SONIA B, PHD
PROGRAM DIRECTOR
CANCER TRAINING BRANCH
CENTER FOR CANCER TRAINING
NATIONAL CANCER INSTITUTE
NATIONAL INSTITUTES OF HEALTH
BETHESDA, MD 20892

KORCZAK, JEANNETTE F, PHD
PROGRAM DIRECTOR
CANCER TRAINING BRANCH
CENTER FOR CANCER TRAINING
NATIONAL CANCER INSTITUTE
NATIONAL INSTITUTES OF HEALTH
ROCKVILLE, MD 20850

Consultants are required to absent themselves from the room during the review of any application if their presence would constitute or appear to constitute a conflict of interest.
